# Clinical Cases in Bellevue Hospital College

**Published:** 1866-02

**Authors:** Frank King


ART. IV—*Clinical Cases in Bellevue Hospital College. Reported by* FRANK KING.

*Fungus Hœmatodes.*—Prof. Hamilton presented I. A., aged 32 years, with a fungus hæmatodes of upper lip and left check, connected with medullary carcinoma at its base, engrafted on a nervus maturna; these are not malignant at an early period of life, but in advanced age they are liable to take on a malignant growth. He had always enjoyed good health till about five weeks ago, when a hemorrhage commenced from the gums, and a small tumor appeared opposite the incisor teeth of upper jaw, which was removed a few weeks after by ligature, but the disease has rapidly returned, and also attacked the nevus on the face. The prognosis of an operation is unfavorable, but unless something is done, it will shortly kill him. The disease is limited, as far as the bone is concerned, to the floor of antrum and immediate neighborhood. With the view of stopping a large portion of its supply of blood, an incision is made from the angles of the mouth a little upwards and outward, previous to the removal of the bone, and the coronary arteries ligated. The diseased bone and the nevus were next extirpated, and the bleeding arrested. He was then removed. During the night he took considerable nourishment, and experienced no difficulty in deglutition, doing well. Next morning Prof. Hamilton made a plastic operation, covering the cavity formed by the removal of the sub. maxillary and the nevus, taking the flap from the left side of the neck, and dressing the wound with lint smeared with simple cerete, over which is placed cotton wool, in order to make the requisite amount of pressure, without strangulation of the tissues with a bandage around the head. He rallied from the second operation, and appeared to be doing well; in the evening he was attacked with a severe pain in the side, and next morning marked dullness was discovered in the plural sac; he gradually sank and died a few days after the operation.

The post-mortem revealed the lungs enlarged and envolved in medullary carcinoma, the plural sac contained blood from the rupture of a vessel, showing that the patient died from internal hemorrhage. He also had what appeared to be a large hydrocele; the most-mortem disclosed it to be a hydrocele disguising a medullary carcinoma of the testicles. The mesenteric glands also were effected with the same disease.

*Excision of Elbow.*—Excision of this joint may be required on account of caries or necrosis of the articulating surfaces of these bones, or when permanent anchylosis exist caused by fracture, or in cases of compound complicated fractures in this joint. The operation is usually successful, a capsular ligament is formed covered with synovial membrane, and a very good joint left, when the general system of the patient has been properly attended to, and when the disease is not extensive, involving the neeessity of cutting into the medullary canal of any of the diseased bones, for in this there is always danger from suppuration and pyæmia. The incision should be made on the posterior surface, on account of the important vessels, nerves and muscles on the anterior, which must not be injured; it may be made in the form of an H or T, or by two longitudinal incisions connected by a transverse incision. The patient, aged about 40, has had disease of this joint for many months. Prof. Wood prefers the H incision, made extensive, for the free escape of pus, from an abscess extending as high up as the insertion of the deltoid muscles, he generally leaves a small portion of the olecranon process, with the periostium, removed for the production of new bone. It is dressed with a leather splint, and the arm placed half way between pronation and supination. Patient doing well.

*Extro-version of Bladder.*—L. D., has a congenital malformation of the bladder and penis, The bladder protudes above the pubis, its posterior wall forms parts of the anterior wall of the abdomen, the surface of the tumor is red, exceedingly irritable, and covered with mucus, thus protecting it somewhat from the atmosphere. The orifice of the ureters open at the inferior part of the tumor, from which the urine constantly dribbles. The penis is split its entire length on the upper surface to the urethra. In this case the testicles and scrotum are well developed; sometimes they are absent, or exist in a rudimentary state. Operations for the removal of this deformity have been made, but generally without success; all that can be done is to keep the parts clean, and the use of a closely-fitting cap of silver or gutta-percha, with a bottle to receive the urine.

